# Effect of norcantharidin on the proliferation, apoptosis, and cell cycle of human mesangial cells

**DOI:** 10.1080/0886022X.2017.1308257

**Published:** 2017-04-10

**Authors:** Kun Ye, Qiaoyu Wei, Zhifeng Gong, Yunfeng Huang, Hong Liu, Ying Li, Xiaomei Peng

**Affiliations:** a Department of Nephrology, The People’s Hospital of Guangxi Zhuang Autonomous Region, Guangxi, China;; b Department of Nephrology, The Second Xiangya Hospital of Central South University, Hunan, China

**Keywords:** Mesangial cell, norcantharidin, proliferation, apoptosis, cell cycle

## Abstract

**Aims:** Norcantharidin (NCTD) regulates immune system function and reduces proteinuria. We sought to investigate the effect of NCTD on proliferation, apoptosis and cell cycle of cultured human mesangial cells (HMC) *in vitro*.

**Methods:** HMC cells were divided into a normal control group, and various concentrations of NCTD group (2.5, 5, 10, 20, or 40 μg/mL). Cell proliferation was determined by 3-(4,5-dimethylthiazol-2-yl)-2,5-diphenyltetrazolium bromide (MTT) assay, apoptosis was detected by Annexin V/propidium iodide (PI) assays, and morphological analysis was performed by Hoechest 33258 staining. Finally, cell cycle was analyzed by flow cytometry.

**Results:** NCTD dose and time dependently inhibits HMC proliferation significantly (*p* < .05). Apoptosis dose and time dependently increased after NCTD treatment. Cell-cycle analysis revealed that the number of cells in the G2 phase increased significantly, whereas the fraction of cells in the S phase decreased, especially 24 h after 5 μg/ml NCTD treatment.

**Conclusion:** NCTD inhibits HMC cell proliferation, induces apoptosis, and affects the cell cycle.

## Introduction

NCTD has been used extensively in a variety of cancers, including hepatocellular carcinoma, breast cancer, and urinary bladder carcinoma,^1–3^ since it could prevent tumorigenesis by inhibiting cell proliferation, inducing apoptosis and the cell cycle arrest, and anti-angiogenic effects.^3–5^

In past studies, NCTD was found to inhibit the proliferation of HK-2 cells induced by albumin *in vitro*, and ameliorated tubulointerstitial fibrosis in diabetic nephropathy models *in vivo*.^6^^,^^7^ Besides, NCTD was also reported to ameliorate the progression of proteinuria-induced renal disease in a rat model.^8^ However, the mechanism of NCTD in renal disease remains unknown, and there was no study about the effect on mesangial cells.

Hyperproliferation and inadequate mesangial cell apoptosis is a common pathway mediating various glomerular diseases, including glomerular sclerosis,^9^ however, whether or not NCTD has inhibitory activities against mesangial cells remains unclear. The purpose of this study was to investigate the effect of NCTD on the proliferation, apoptosis and cell cycle of cultured human mesangial cells *in vitro*.

## Materials and methods

### Materials

NCTD was purchased from Sichuan Sunnyhope Pharmaceutical Co., LTD (Sichuan, China). Fetal bovine serum (FBS) and RPMI-1640 were purchased from Gibco, Inc. (Grand Island, NY). Dimethyl sulfoxide and MTT were purchased from Beijing Solarbio Science & Technology Co., Ltd (Beijing, China). Trypsin was purchased from Life Technologies (Madison, WI). The Annexin V-FITC/PI Apoptosis Detection Kit and Cell Cycle Detection Kit were purchased from Nanjing Keygen Technology Development Co., Ltd (Nanjing, China).

### Cell culture

Human mesangial cells (HMC) were obtained from Professor Wen-ge Li of the China-Japan Friendship Hospital. HMC cells were cultured in RPMI-1640 supplemented with 10% FBS, 100 U/mL penicillin, and 100 mg/mL streptomycin at 37 °C in a humidified atmosphere containing 5% CO_2_.

### MTT assay

Cells were seeded in a 96-well tissue culture plate at a density 1 × 10^4^ cells/well. HMC cells were incubated with varying concentrations of NCTD (2.5, 5, 10, 20, or 40 μg/mL) for 12, 24, or 48 h. Untreated cells served as the control group. After treatment, MTT was added to each well at a final concentration of 5 mg/mL, and the cells were further incubated at 37 °C for 4 h. Dimethyl sulfoxide (100 μL) was added to each well after removing the medium. After shaking the plates for 5 min, the absorbance of the mixture was measured at 490 nm using a microplate enzyme-linked immunosorbent assay (ELISA) reader (GF-M3000, Shandong, China). The results were calculated as follows: inhibition rate = ([mean control absorbance − control blank absorbance] – [mean experimental absorbance − experimental blank absorbance])/(mean control absorbance − control blank absorbance) × 100%. The concentration that caused 50% growth inhibition (IC_50_) was calculated and used in additional assays.

### Apoptosis assays

Based on the results of the MTT assay, the HMC cells were divided into the following four groups: control, NCTD (2.5 μg/mL), NCTD (5 μg/mL), and NCTD (10 μg/mL). Cells were treated as described for 12 and 24 h.

The apoptosis rate was evaluated using the Annexin V-FITC/PI Apoptosis Detection kit according to the instructions from the manufacturer. The cells were seeded into 6-well tissue culture plates (4 × 10^5^ cells/well). Following treatment, the cells were collected, washed with PBS, and resuspended in 500 μL binding buffer. Then, 5 μL Annexin V-FITC and 5 μL PI were added to the buffer and incubated at room temperature for 15 min in the dark. Cells were analyzed by flow cytometry (BD FACSCanto) within 1 h.

### Hoechest 33258 staining assay

In order to investigate the cell apoptosis, morphological analysis was performed by Hoechest 33258 staining. Replicate cultures of 1 × 10^6^ HMC cells per well were plated in a 6-well plate. Following the treatment with different concentrations of NCTD, the cells were incubated with 5 μL of Hoechst 33258 (Beyotime, Nanjing, China) solution per well at 37 °C for 10 min, followed by observation under a fluorescence microscope. Strong fluorescence can be observed in the nuclei of apoptotic cells, while weak fluorescence was observed in non-apoptotic cells.

### Cell-cycle analysis

Cells were seeded in a 6-well tissue culture plate (4 × 10^5^ cells/well) and NCTD as described in the section "Apoptosis assays". After treatment, the cells were collected and washed with PBS. RNase A solution (100 μL) was added, and cells were incubated for 30 min at 37 °C. Finally, 400 μL PI was added and incubated for 30 min at room temperature. The DNA content was detected by flow cytometry. The data were analyzed by Cell Quest software (Becton Dickinson, Franklin Lakes, NJ). The percentage of cells in the G1 phase, the S phase, and the G2 phase was analyzed.

### Statistical analysis

Statistical analysis was performed with SPSS version 13.0 (SPSS Inc., Chicago, IL), and all quantitative data are presented as the mean ± standard deviation. Differences between two groups were compared using Student’s *t*-test when they had a normal distribution. A one-way analysis of variance (ANOVA) was used to compare data among groups when they had a normal distribution and homogeneous variances. A *p* value less than 0.05 was considered statistically significant.

## Results

### Effect of NCTD on HMC cell proliferation

**Figure 1.**
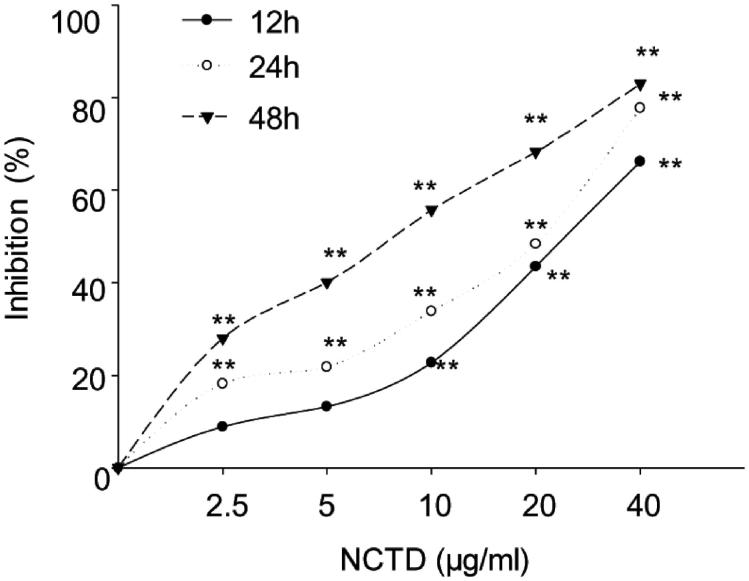
Effect of NCTD on the proliferation of HMC cells, as determined by MTT assay. *p < .05, **p < .01 indicates a significant difference versus the control group.

MTT assay demonstrated that NCTD could inhibit cell proliferation on HMC cells, and the inhibitory rate dose and time dependently increased following NCTD treatment (*p* < .05). As presented in Figure 1, after 12 h, cell growth was inhibited 45.17 ± 13.36% and 65.88 ± 16.62% versus control when treated with 20 μg/mL and 40 μg/mL NCTD (*p* < .01). After 24 h, HMC cell growth was inhibited 52.57 ± 13.87% and 76.20 ± 11.33% versus control when treated with 20 μg/mL and 40 μg/mL NCTD (*p* < .01). After 48 h, HMC cell growth was inhibited 53.17 ± 8.69%, 64.94 ± 11.27%, and 81.55 ± 8.51% with 10 μg/mL, 20 μg/mL, and 40 μg/mL NCTD (*p* < .01).

According to the above results, the inhibitory rate after 48 h NCTD treatment and with the concentration of 20 μg/mL and 40 μg/mL NCTD was close to or more than 50%, therefore, we chose to use 2.5, 5, and 10 μg/mL NCTD treated for only 12 h and 24 h for additional studies.

### Effect of NCTD on apoptosis of HMC cells

**Figure 2.**
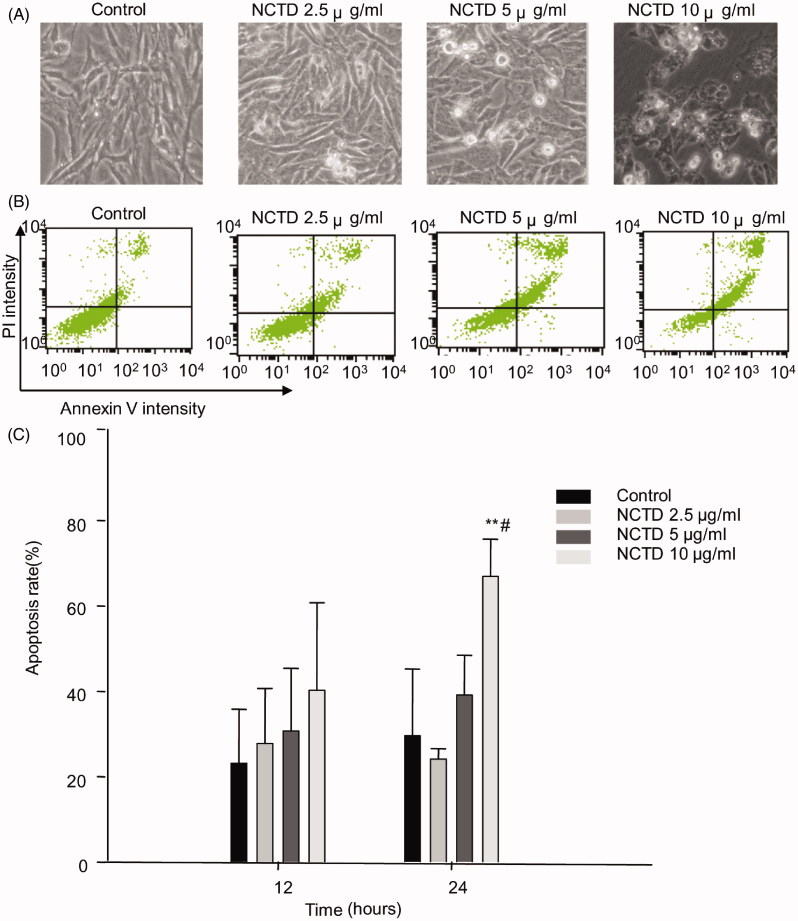
Effect of NCTD on the apoptosis of HMC cells. NCTD induced morphological changes of HMC cells (A). NCTD induced apoptosis of HMC cells (B). *p < .05, **p < .01 indicates a significant difference versus the control group, ^#^*p* < .05, ^##^*p* < .01 indicates a significant difference versus the 12 h group (C).

We used the annexin V and PI double staining kit to quantify HMC cell apoptosis. The percentage of specific cell populations at various stages of apoptosis is shown in Figure 2.

After 12 h, the apoptosis rate in the control group was 23.22 ± 12.64%. NCTD treatment dose dependently increased the rate of apoptosis; however, there was no difference compared to the control group (*p* > .05).

After 24 h, the rate of apoptosis in the control group was 29.62 ± 15.60%. In contrast, the apoptosis rate dose dependently increased following NCTD treatment. Apoptosis was significantly increased after treatment with 10 μg/mL NCTD (66.95 ± 8.7%) compared with the control (*p* < .01 versus control, and *p* < .05 versus 12 h treatment).

### Effect of NCTD on cytomorphology of HMC cells

**Figure 3.**
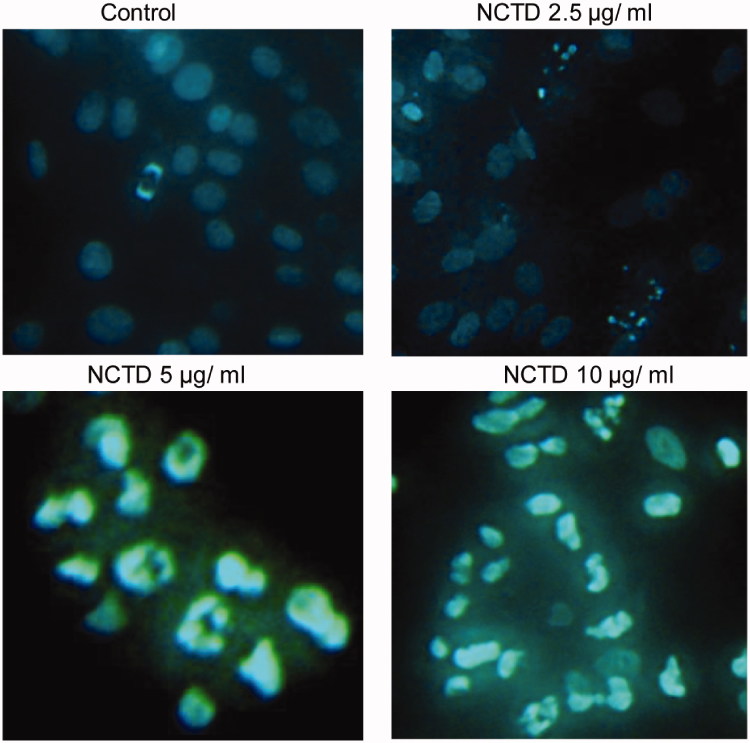
Effect of NCTD on cytomorphology of HMC cells (×400).

As shown in Figure 3, the bodies of apoptotic cells shrinked in volume and became round, and the concentration of cell nucleus was observed, and cell nucleus became white after stained by Hoechst 33258 under a fluorescent microscope. HMC treated with NCTD showed significant chromatin condensation, cellular shrinkage, apoptotic bodies, and cytoplasmic condensation. These cellular changes were typically redundant characteristics of apoptosis. HMC without NCTD maintained normal chromatin patterns and cell size.

### Effect of NCTD on the cell cycle in HMC cells

**Figure 4.**
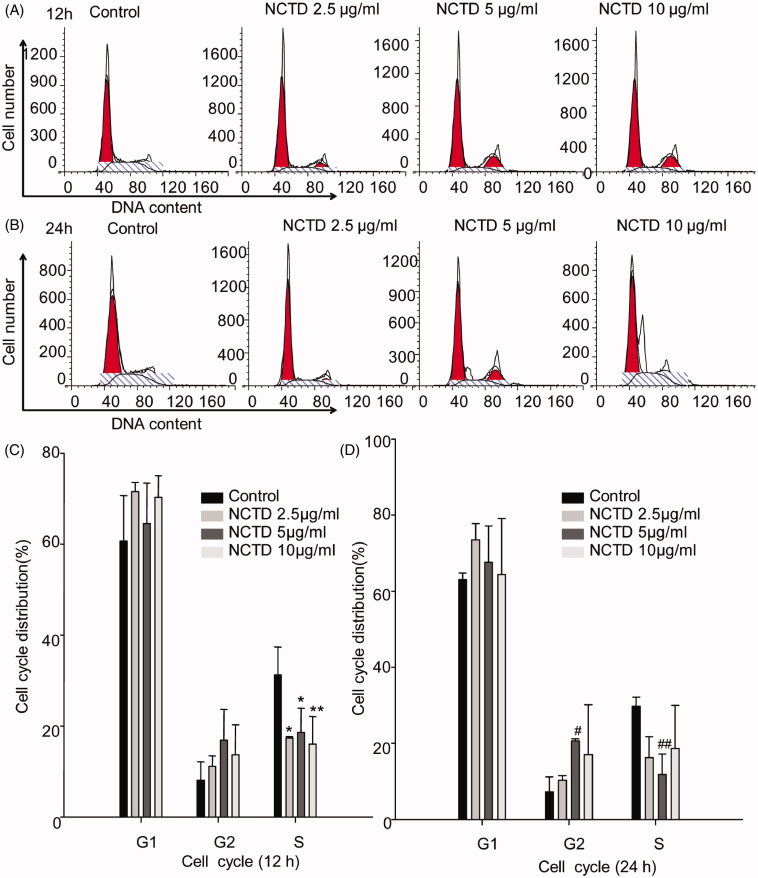
Effect of NCTD on the cell cycle in HMC cells. Distribution of cell cycle for HMC cells after treated with various concentrations of NCTD for 12 h and 24 h (A, B). *p < .05, *p < .01 indicates a significant difference versus the control group (C). ^#^*p* < .05, ^##^*p* < .01 indicates a significant difference versus the control group (D).

To explore whether NCTD-induced apoptosis was associated with cell cycle arrest, we detected the cell cycle distribution of HMC cells using flow cytometry to analyze cellular DNA content. As shown in Figure 3, there was a significant decrease in the percentage of HMC cells with 2.5 μg/mL and 5 μg/mL NCTD after 12 h treatment in the S phase versus control (*p* < .05), while there was further decrease 10 μg/mL NCTD treatment(*p* < .01). After 24 h treatment, there was increase in the percentage of HMC cells in the G2 phase versus control (*p* < .05), whereas the percentage of cells with 5 μg/mL NCTD in the S phase decreased (*p* < .01) (Figure 4).

## Discussion

Cantharidin (CTD), an active compound found in blister beetles, is used as an antitumor therapeutic in many cancers. However, due to its significant adverse effects, its clinical use is limited.^10^^,^^11^ Recently, its demethylated analog, norcantharidin (NCTD), was shown to have reduced cytotoxicity, and could have clinical applications, especially in cancer treatment.^1–3^

Besides its antitumor function, NCTD also regulates immune function, specifically leukocytes.^12^ Further, NCTD reduces proteinuria, and there may be at least three mechanisms for NCTD to ameliorate proteinuria-induced renal disease: attenuation of proteinuria, inhibition of interstitial inflammation, and reduction of intrarenal fibrosis.^4^^,^^6^^,^^7^ Previous studies indicated that NCTD attenuated renal interstitial fibrosis and inhibited HK-2 cell proliferation.^13^^,^^14^ A recent study showed that NCTD exerts an anti-fibrosis effect via inhibition of PP2Ac expression.^15^ Most of the studies indicate that NCTD is a protect agent for tubulointerstitial fibrosis.^16^ However, the effect of NCTD on mesangial cells has not been reported.

This study was to investigate the effect of NCTD on mesangial cell proliferation and apoptosis using MTT and Annexin V/propidium iodide (PI) assays, and by analyzing cell cycle by flow cytometry. Combined, our data provide support for the use of NCTD in glomerular disease.

MTT assays show that NCTD could significantly greater inhibition of proliferation than control cells. Further, this inhibition occurred in a dose- and time-dependent manner. The result is the same as the study by Li, which showed that NCTD could inhibit HK-2 cell proliferation.^6^

Apoptosis, or programmed cell death, is a complicated process that involves multiple genes. Interestingly, apoptosis can be induced during cell cycle arrest.^17^ In our study, we analyzed changes in apoptosis and cell cycle by flow cytometry. Our results indicated that NCTD treatment dose and time dependently induced HMC cell apoptosis, and the result of Hoechst 33258 confirmed that the cell was apoptotic. These data were in accordance with a study by Ding,^18^ which showed that NCTD could induce apoptosis in many cell types, including HL-60 and DU145 cells.^19^^,^^20^ The results matched with the outcome with MTT assays.

NCTD was previously shown to induce cell-cycle arrest through G2 arrest.^21^^,^^22^ However, some groups have also suggested that NCTD inhibits the cell cycle at the G1 or the S phase.^8^^,^^23^ As we know, there were two important stages in the cell cycle, which were the G2 phase to the M phase and the G1 phase to the S phase. The G2 phase is the period of the cell cycle following DNA amplification, just prior to cells entering mitosis. If the cell cycle is arrested during the G2 phase, growth is inhibited. Therefore, we used flow cytometry to analyze the DNA content of the cells after NCTD treatment. Our study showed that the G2 phase arrested and the S phase decreased; therefore, it could lead to the DNA synthesis decreased. Thus, our data indicate that the inhibitory effect of NCTD may correlate with changes in the cell cycle, and these data were also in accordance with the results of the apoptosis assay. The results were more obvious after 5 ng/mL NCTD treatment for 24 h, the mechanism of which was still need to be confirmed in the follow-up experiment.

In conclusion, this study showed that NCTD can inhibit the proliferation and increase the apoptosis of HMC cells. Further, the effects of NCTD may be mediated by the inhibition of HMC cell proliferation, disturbances in the cell cycle, decreased DNA synthesis, and induction of cell apoptosis. Our results suggest that NCTD may represent a novel therapeutic for the treatment of mesangial proliferative glomerulonephritis. However, the mechanism is still unclear, so further studies should be performed to confirm for the mechanism of NCTD in HMC cells. Therefore, we are currently conducting cell experiments and animal experiments to confirm the effect of NCTD on mesangial cells and its effects on animal models, and we hope to find more evidence to confirm the effect of NCTD on mesangial cells.
